# Prevention of Transition from Acute Kidney Injury to Chronic Kidney Disease Using Clinical-Grade Perinatal Stem Cells in Non-Clinical Study

**DOI:** 10.3390/ijms25179647

**Published:** 2024-09-06

**Authors:** Agne Gryguc, Justinas Maciulaitis, Lukas Mickevicius, Arvydas Laurinavicius, Neringa Sutkeviciene, Ramune Grigaleviciute, Vilma Zigmantaite, Romaldas Maciulaitis, Inga Arune Bumblyte

**Affiliations:** 1 Department of Nephrology, Medical Academy, Lithuanian University of Health Sciences, 44307 Kaunas, Lithuania; agne.gryguc@lsmuni.lt (A.G.); ingaarune.bumblyte@lsmu.lt (I.A.B.); 2 Hospital of Lithuanian University of Health Science, 50161 Kaunas, Lithuania; 3 Institute of Cardiology, Lithuanian University of Health Sciences, 44307 Kaunas, Lithuania; justinas.maciulaitis@lsmu.lt; 4 Institute of Physiology and Pharmacology, Lithuanian University of Health Sciences, 44307 Kaunas, Lithuania; 5 Department of Urology, Lithuanian University of Health Sciences, 44307 Kaunas, Lithuania; lukas.mickevicius@lsmu.lt; 6 National Center of Pathology, Affiliate of Vilnius University Hospital Santaros Klinikos, 08661 Vilnius, Lithuania; arvydas.laurinavicius@vpc.lt; 7 Large Animal Clinic, Veterinary Academy, Lithuanian University of Health Sciences, 44307 Kaunas, Lithuania; neringa.sutkeviciene@lsmuni.lt; 8 Biological Research Center, Veterinary Academy, Lithuanian University of Health Sciences, 44307 Kaunas, Lithuania; ramune.grigaleviciute@lsmu.lt (R.G.); vilma.zigmantaite@lsmu.lt (V.Z.); 9 Faculty of Medicine, Medical Academy, Lithuanian University of Health Sciences, 44307 Kaunas, Lithuania

**Keywords:** perinatal stem cell, prevention, acute kidney injury, chronic kidney disease

## Abstract

Acute kidney injury (AKI) is widely recognized as a precursor to the onset or rapid progression of chronic kidney disease (CKD). However, there is currently no effective treatment available for AKI, underscoring the urgent need for the development of new strategies to improve kidney function. Human placental mesenchymal stromal cells (hpMSCs) were isolated from donor placentas, cultured, and characterized with regard to yield, viability, flow cytometry, and potency. To mimic AKI and its progression to CKD in a rat model, a dedicated sensitive non-clinical bilateral kidney ischemia-reperfusion injury (IRI) model was utilized. The experimental group received 3 × 10^5^ hpMSCs into each kidney, while the control group received IRI and saline and the untreated group received IRI only. Urine, serum, and kidney tissue samples were collected over a period of 28 days. The hpMSCs exhibited consistent yields, viability, and expression of mesenchymal lineage markers, and were also shown to suppress T cell proliferation in a dose-dependent manner. To ensure optimal donor selection, manufacturing optimization, and rigorous quality control, the rigorous Good Manufacturing Practice (GMP) conditions were utilized. The results indicated that hpMSCs increased rat survival rates and improved kidney function by decreasing serum creatinine, urea, potassium, and fractionated potassium levels. Furthermore, the study demonstrated that hpMSCs can prevent the initial stages of kidney structural fibrosis and improve kidney function in the early stages by mitigating late interstitial fibrosis and tubular atrophy. Additionally, a robust manufacturing process with consistent technical parameters was established.

## 1. Introduction

Acute kidney injury (AKI) is a condition [1] that frequently occurs in hospitalized patients with severe illnesses and also as a postoperative complication [1,2,3,4,5]. Although most AKI patients recover, their recovery is highly reliant on factors such as baseline kidney function, severity, duration, and etiology [6,7]. Despite the possibility of AKI reversal, approximately 15–20% of AKI patients progress to end-stage chronic kidney disease (CKD) [8,9,10,11]. CKD is defined as abnormalities of kidney structure or function by KDIGO (Kidney Disease: Improving Global Outcomes) [12]. In addition, the risk of developing CKD is 43 times higher in AKI patients than in non-AKI patients during the first six months after an AKI episode [13]. The increasing number of patients with CKD and the associated high morbidity and mortality rates represent a significant problem for the general public [8,14,15,16,17]. In Europe, 716.7 inhabitants per million live births are treated with renal replacement therapy (RRT) for end-stage renal injury. Many treatments have been attempted to slow or stop CKD progression, but there is currently no effective or timely treatment available. Therefore, it is essential to develop new strategies to maintain kidney function [18,19].

Mesenchymal stromal/stem cells (MSCs) are a type of mesodermal non-embryonic cells that are derived from adult cells of the mesodermal germ layer or undifferentiated stem cells. Due to their protective and regenerative properties, these cells have garnered attention for their potential in treating kidney injury [20,21,22,23,24,25,26]. Allogeneic human placenta-derived mesenchymal stromal cells (hpMSCs) may present a viable solution for preventing and treating AKI-to-CKD conversion [27]. These cells exhibit characteristics of both pluripotent embryonic stem cells and multipotent mesenchymal stem cells [27,28,29,30,31,32,33,34,35,36,37] and have demonstrated potential therapeutic capabilities in both in vitro and in vivo settings [28]. The placenta has been utilized for cell isolation and preclinical testing [25,26,28,38,39,40,41,42,43,44]; however, the use of non-standardized manufacturing approaches has hindered its effective clinical translation. The ongoing challenge of translating and reproducing basic scientific findings to clinical setting remains a significant issue [30]. The use of Current Good Manufacturing Practice (cGMP)-grade preparation of active cell-based medicinal products can significantly improve the standardization and characterization of the manufacturing process. By applying a cGMP-specific approach that involves critical quality attributes and process parameters in a preclinical setting, this can be carried over to subsequent clinical trials [45].

To the best of our knowledge, this study is the first to fully standardize cGMP-grade hpMSCs of placental origin with highly proliferative and potent features and to evaluate them in the treatment of AKI and prevention of CKD in a non-clinical model.

## 2. Results

### 2.1. Donor Variability

**Figure 1:**
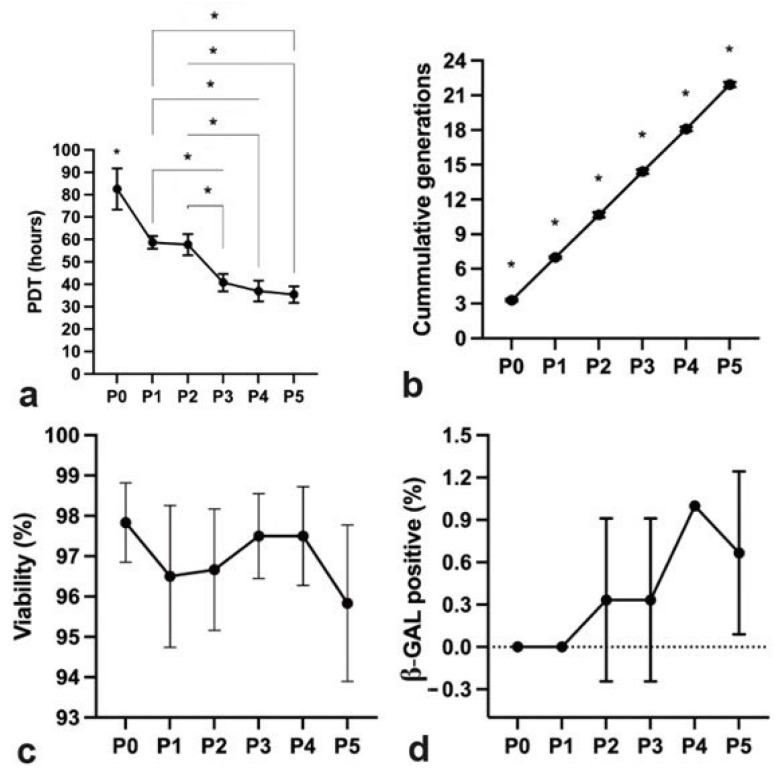
Characteristics and consistency of hpMSCs across passages. (**a**) Population doubling times after manufacturing optimization: P0 was notably shorter than the other passages (* *p* < 0.05); P3, P4, and P5 were significantly reduced compared with P2 or P3. (**b**) Uniformity of cumulative generations between consecutive distinct manufacturing runs (* *p* < 0.05). (**c**) Consistent inter-donor hpMSC viability (95.83 ± 1.94%). (**d**) Invariable senescence of cells up to P5, with almost undetectable levels at the drug-product stage.

Donor variability can affect the initial stages of hpMSCs preparation, but according to the process verification data (Supplementary Materials Table S1), the cell growth kinetics were uniform across the population of hpMSCs. To assess the quality of hpMSCs and donor suitability, cell population doubling and identity were critical indicators which were further supported by a peripheral blood mononuclear (PBMC) proliferation assay as an additional parameter for potency qualification. The results showed that the three batches of hpMSCs derived from different donors exhibited the predetermined specifications, indicating that the observed variation did not have any biological impact (as shown in Figure 1).

### 2.2. hpMSCs Exhibit Efficient Proliferation Capacity

**Figure 2:**
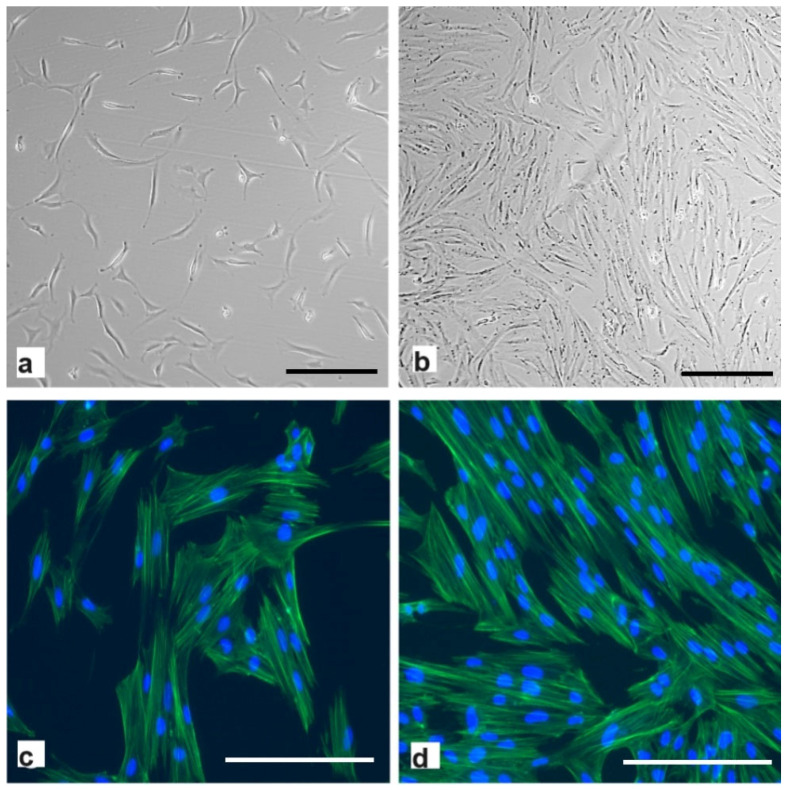
Light microscopy images of thawed second-passage human placenta-derived mesenchymal stromal cells were taken (**a**) 24 h and (**b**) 5 days post-seeding. Scale bar: 100 μm. Representative images of actin filaments (phalloidin) and nuclei (Hoechst 33258 stain) at (**c**) 24 h and (**d**) 5 days. Scale bar: 200 μm.

The hpMSCs exhibited an efficient proliferation capacity, with morphologies resembling the round spindle shape of mesenchymal stem cells (Figure 2). The cell shape remained relatively unchanged at the beginning of the culture or at confluence before passage six.

The population-doubling times at P0 were lower than those of other passage cells (*p* < 0.05) (Figure 1). P1 and P2 were comparable; however, P3, P4, and P5 were significantly shorter than P2 and P3 (*p* < 0.05). The cumulative generations between passages were significantly different from those of the previous passage (*p* < 0.05).

The human mesenchymal stem cells (hpMSCs) exhibited a high level of viability, at 95.83% ± 1.94%. These cells were harvested and cryopreserved at a concentration of 1 × 106 cells/mL.

During the early stages of hpMSC preparation (up to P5), senescent cells were not observed. However, as hpMSCs in culture did reach senescence at later passages, only minimal levels of senescent cells were present at the drug-product stage.

### 2.3. GMP-Grade Production of hpMSCs

The scarcity of cultured MSCs can be partially attributed to the variability in the original MSC populations, differences between donors, and the range of sources from which MSCs are initially obtained [46].

To demonstrate the non-variability between different placental tissue donors and their impact on the resultant drug product, the medicinal prototype-preparation process was verified by the principles outlined in the EU-GMP Annex 15 and EMA guidelines [47].

Additionally, process control and release criteria were used to assess MSC consistency. New batches derived from different donors were produced to confirm the robustness of this process. The drug-product batches were manufactured by adhering to specific IPCs and release specifications. This approach demonstrated that donor variability did not adversely affect the quality of the resulting drug product by ensuring that MSCs from different placental donor tissues met the predefined quality specifications.

No bacterial growth or mycoplasma was observed in the cell supernatants throughout the process. Kinetic chromogenic endotoxin analysis indicated that endotoxin levels were below the acceptable limit of 5 EU/kg.

**Figure 3:**
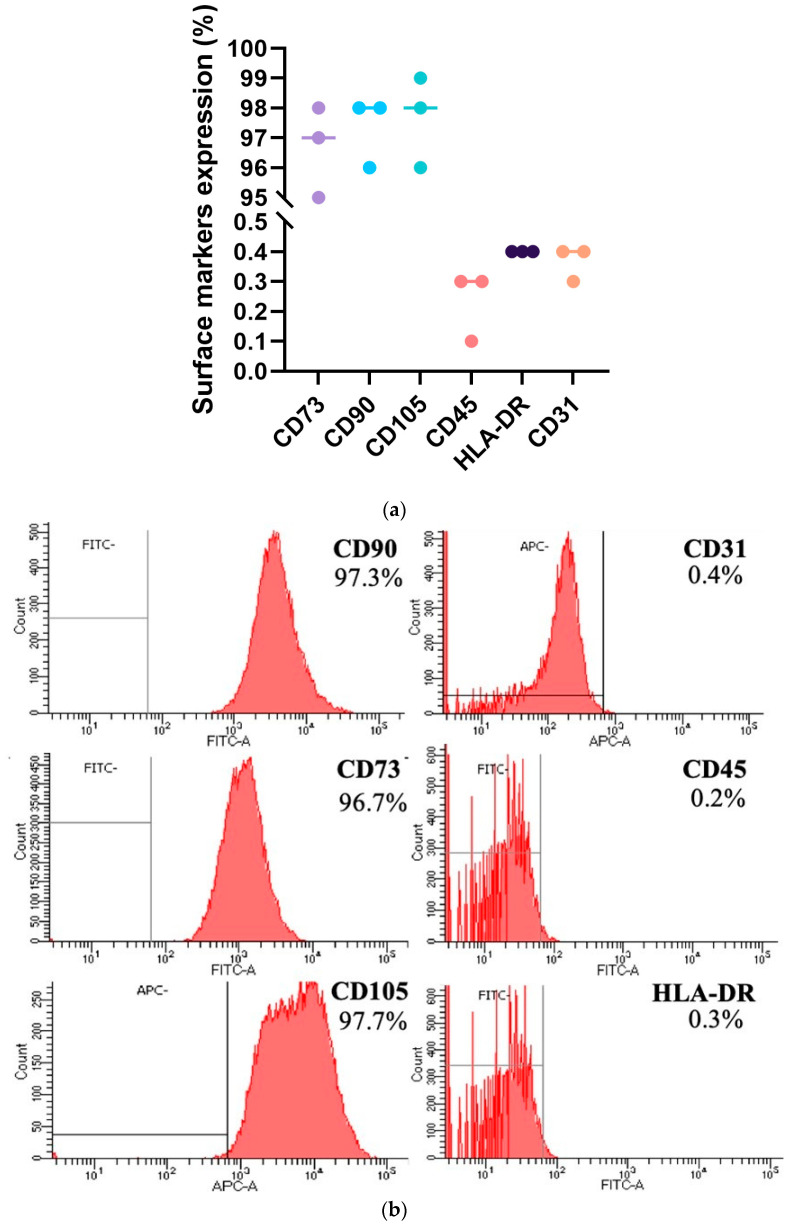
(**a**) Flow cytometry data of MSCs derived from the placenta of three donors indicate uniform MSC purity across samples from different donors. (**b**) More than 95% of the cells from each donor consistently expressed CD73, CD90, and CD105, confirming their mesenchymal identity. Endothelial (CD31), hematopoietic (CD45), and antigen-presenting (HLA-DR) markers were present in less than 1% of the cells, demonstrating minimal non-mesenchymal lineage presence and a repeatable manufacturing process.

**Figure 4:**
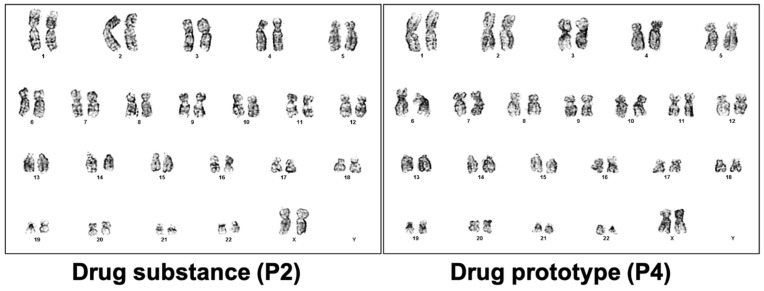
Representative karyotyping results of MSCs’ stability at drug-substance and drug-product stages. Normal complete karyogram (46, XX) of samples from distinct manufacturing stages.

Immunophenotypic characterization revealed that more than 95 percent of the formulated drug product exhibited CD73, CD90, and CD105 expression and less than 1 percent exhibited CD31, CD45, and HLA-DR expression (Figure 3). Passage of in vitro-expanded hpMSCs did not affect the expression profile of these markers. G-banding karyotype analyses revealed that the hpMSCs had a stable 46 chromosome (XX) karyotype (Figure 4). In addition, hpMSCs exhibited multilineage differentiation potential in adipogenic, chondrogenic, and osteogenic culture medias.

### 2.4. Potency Mode of Action of hpMSCs

**Figure 5:**
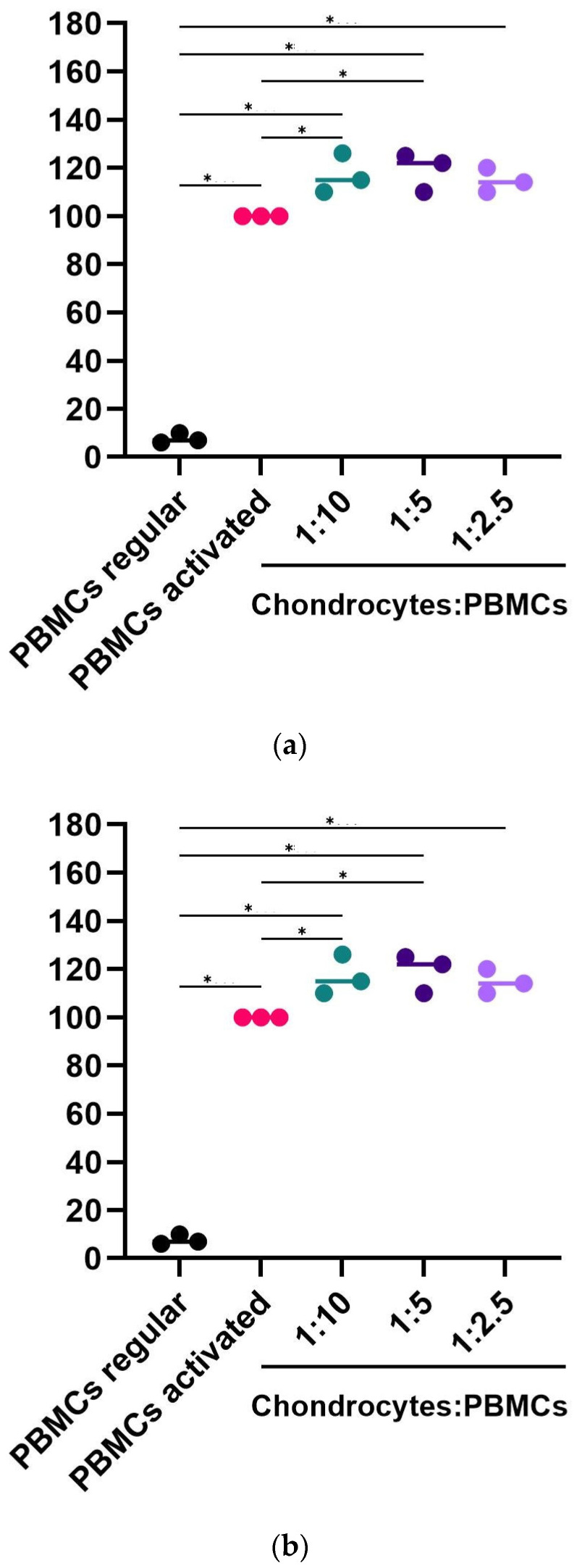
hpMSCs consistently inhibited lymphocyte proliferation. (**a**) PBMCs (5 × 10^5^) were stimulated with PHA and ionomycin in the presence of increasing concentrations of hpMSCs. (**b**) Chondrocytes were used as the negative controls (n = 3). Consistent potency results between separate manufacturing runs are expressed as the percentage of proliferation, with the positive control at 100%. The results are presented as mean ± SEM (* *p* < 0.05).

The co-culture of hpMSCs with PBMCs stimulated with PMA/ionomycin resulted in dose-dependent inhibition of PBMCs’ proliferation (*p* < 0.05) (Figure 5). A negative control of PBMCs’ co-culture with chondrocytes did not affect T-cell proliferation. These findings indicate that hpMSCs can exert an immunosuppressive effect on T cells in a dose-dependent fashion.

**Figure 6:**
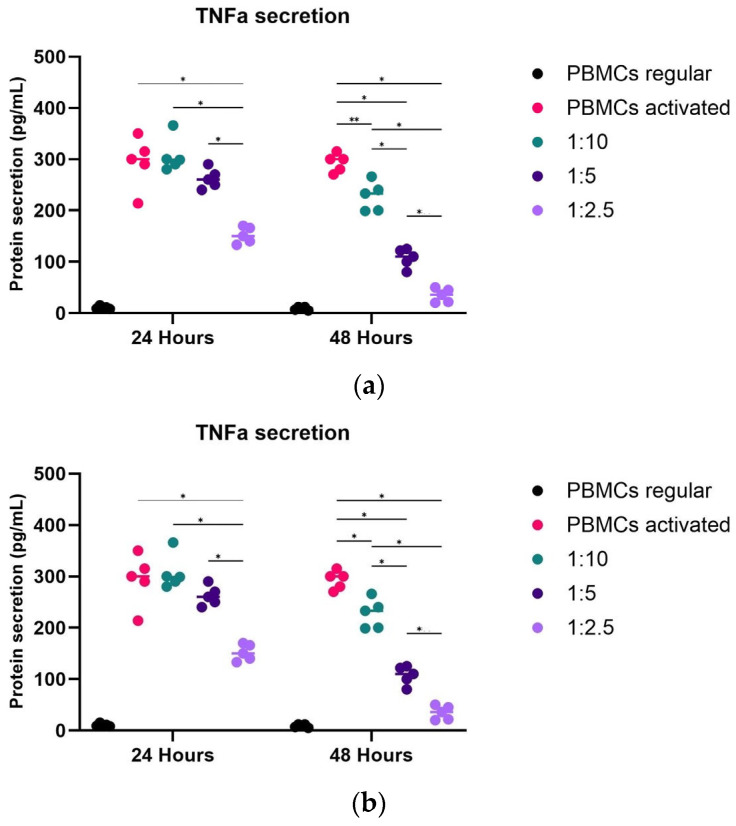
hpMSCs that inhibited the secretion of TNF-α and IL-6 PBMC (5 × 10^5^) were stimulated with PMA and ionomycin in the presence of increasing concentrations of hpMSCs. Consistent results between distinct drug product manufacturing runs are expressed as protein secretion in pg/mL of (**a**) TNF-α and (**b**) IL-6. The results are shown as mean ± SEM. * *p* < 0.05, ** *p* < 0.01.

hpMSCs exerted dose-dependent inhibition of TNF-α and IL-6 production (Figure 6). hpMSCs had superior TNF-α and IL-6 protein secretion-suppressing capabilities (*p* < 0.05) and improved potency in diminishing inflammation in all groups tested. The highest inhibitory effect was evident in the 1:2.5 group after 48 h. TNF-α and IL-6 levels in cultures with PSCs and unstimulated PBMC, chondrocytes, or PBMC alone were below the detection limit of the assay.

### 2.5. Survival Rate

**Figure 7:**
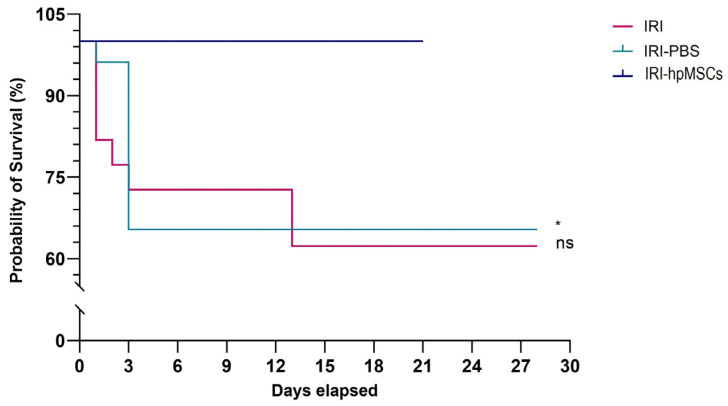
Survival in the experimental groups until day 28. In the cell group, 100% of rats survived. IRI-PBS (green line) vs. hpMSCs (blue line), * *p* < 0.05. IRI (red line) vs. IRI-hpMSCs (blue line) and ns (non significant) (*p* = 0.054).

The results of this study revealed that all the animals in the hpMSC group survived until the end of the 21-day period (Figure 7). In contrast, the IRI and IRI-PBS groups had the lowest survival rates, with the highest mortality occurring within the first three days of the study. At the end of the study, the survival rates in the IRI and IRI-PBS groups were 68% and 65%, respectively, significantly lower than the 100% survival rate observed in the hpMSC group.

### 2.6. Kidney Function

**Figure 8:**
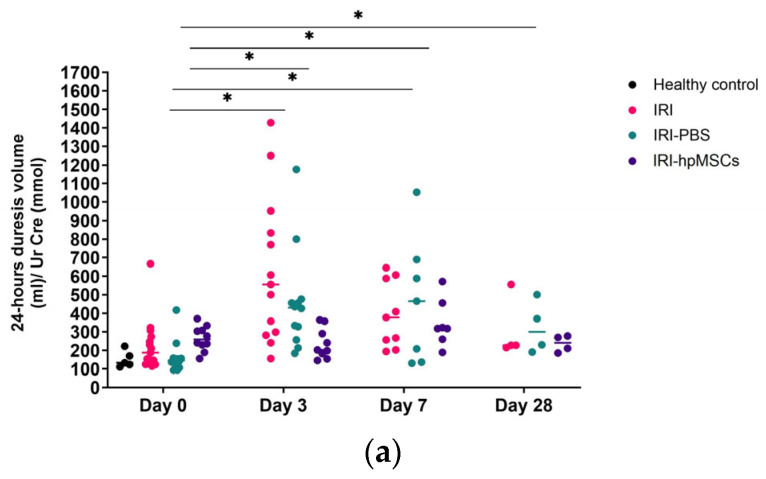

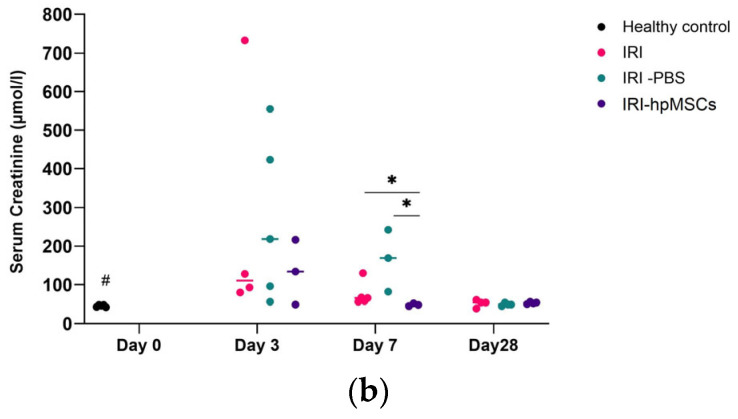
Kidney function (median values are shown in figures). (**a**) Kidneys retained normal diuresis in the cell group. Total diuresis volume over 24 h in the experimental groups. IRI-D0 vs. D3 and D7; IRI-PBS-D0 vs. D3, D7, and D28, * *p* < 0.05; IRI-hpMSCs-D0 vs. D3, D7, and D28, ns. (**b**) Kidneys attenuated the increase in serum creatinine level in the cell group. Serum creatinine levels in the experimental groups. IRI-D7 vs. IRI-hpMSCs-D7; IRI-PBS-D7 vs. IRI-hpMSCs -D7, * *p* < 0.05; healthy controls vs. IRI-D3, D7 and D28; healthy controls vs. IRI-PBS-D3, D7 vs. D28, # *p* < 0.05; healthy controls vs. D3, D7, and D28, ns.

hpMSCs significantly improved kidney function by decreasing creatinine, urea, and fractionated potassium levels in serum on days 3 and 7 compared to the surviving rats in the IRI and IRI-PBS groups (Figure 8, Supplementary Materials Figures S1–S3). Additionally, hpMSCs improved kidney function by maintaining normal 24 h diuresis volume and volume change; creatinine levels in urine, serum, and urine potassium levels; 24 h urine potassium change; and fractionated sodium levels throughout the 21–28-day study period, in comparison to the death-censored control groups. These results were consistent with the changes in creatinine and urea in 24 h urine samples In a study assessing the effects of hpMSCs on serum creatinine level, the IRI-hpMSC group exhibited better renal function compared to both IRI and IRI-PBS groups (Figure 8). On day 3 after IRI, the IRI-hpMSC group showed superior renal function compared to the other groups. Furthermore, the IRI-hpMSC group experienced a more rapid normalization of creatinine levels on day 7 than the IRI and IRI-PBS groups. On day 7 after IRI, the serum creatinine level in the IRI-hpMSC group was nearly 3.5 times lower (*p* = 0.014) than that in the IRI-PBS group and 1.6 times lower (*p* = 0.028) than that in the IRI group. The results revealed that urine urea levels remained lower in the IRI group on day 28 but returned to normal in the IRI-hpMSC group. On day 7, a non-significant trend was observed, with the lower creatinine clearance in IRI-PBS group compared to the surviving rats in the IRI-hpMSC group (*p* = 0.66). (Supplementary Materials Figure S2) On day 28, creatinine clearance was significantly higher in the death-censored IRI group than in the IRI-hpMSC group (*p* < 0.05).

The higher creatinine clearance in the IRI group than that in the healthy control group on day 28 was interpreted as hyperfiltration, a hallmark of acute kidney injury. These results suggest that hpMSCs can restore creatinine clearance more rapidly than control groups. Details of the results are provided in the Supplementary Materials.

### 2.7. Kidney Morphology

**Figure 9:**
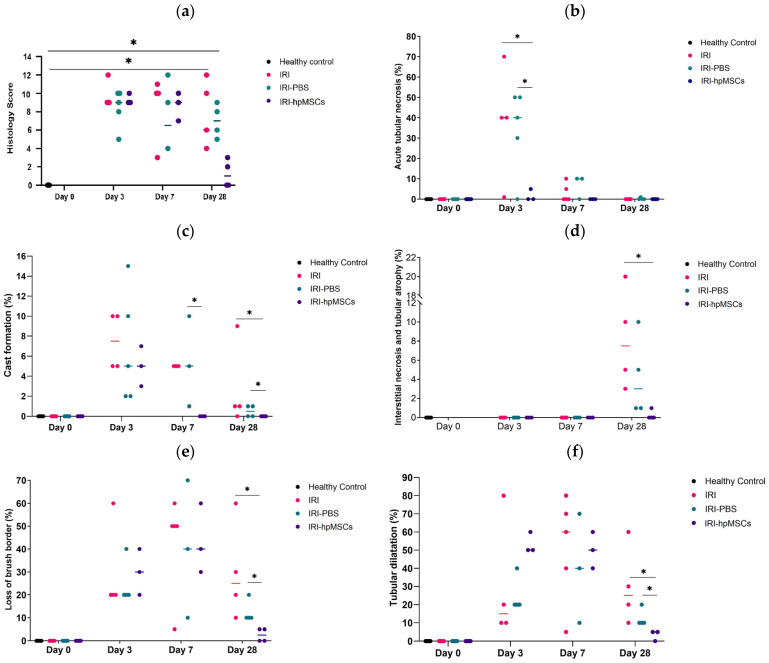

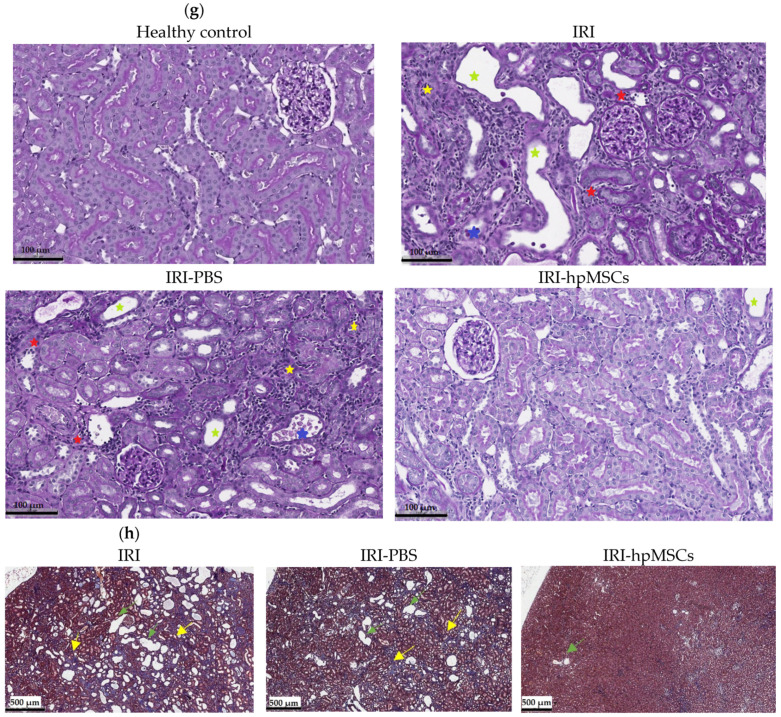
Kidney histology in the experimental groups. hpMSCs reduce structural damage to kidneys and prevent chronic injury. (**a**) Kidney injury scores in the experimental groups. Healthy controls (day 0) vs. IRI-D28, IRI-PBS-D28, * *p* < 0.05. Healthy controls (day 0) vs. IRI-hpMSCs, ns. (**b**) Acute tubular necrosis in the experimental groups. IRI-D3, IRI-PBS-D3 vs. IRI-hpMSCs-D3, * *p* < 0.05. (**c**) Cast formation in experimental groups. IRI-PBS-D7 vs. IRI-hpMSCs-D7; IRI-D28, IRI-PBS-D28 vs. IRI-hpMSCs-D28, * *p* < 0.05. (**d**) Interstitial necrosis and tubular atrophy in experimental groups. IRI-D28 vs. IRI-hpMSCs-D28, * *p* < 0.05. (**e**) Loss of brush border in the experimental groups. IRI-D28, IRI-PBS-D28 vs. IRI-hpMSCs-D28, * *p* < 0.05. (**f**) Tubular dilatation in the experimental groups. IRI-D28, IRI-PBS-D28 vs. IRI-hpMSCs-D28, * *p* < 0.05. (**g**) PAS staining of kidney sections from healthy control, IRI, IRI-PBS, and IRI-hpMSCs animals (day 28), showing a higher degree of leukostasis (red asterisk), tubular dilatation (green asterisk), loss of brush border, cast formation, interstitial necrosis, tubular atrophy (yellow asterisk), debris, or necrotic tissues (blue asterisk) in the control groups than in the normal and cell groups. Bars = 100 µm. (**h**) Masson’s trichrome staining of kidney sections from IRI, IRI-PBS, and IRI-hpMSCs animals (day 28), showing a higher degree of interstitial fibrosis (yellow arrow) and tubular atrophy (green arrow) in the control groups than in the normal and cell groups. Bars = 500 µm.

The results of the histological study demonstrated that perinatal stem cells provided long-lasting protection against kidney injury (Figure 9). On day 3, the IRI group showed widespread acute tubular necrosis, whereas the IRI-hpMSC group showed varied acute tubular necrosis without significant coagulative necrosis. The IRI-hpMSC group exhibited significantly lower levels of acute tubular necrosis on day 3 compared to the IRI (*p* = 0.047) and IRI-PBS (*p* = 0.028) groups. Comparatively, the cell group had a distinct tendency toward a smaller ATN region, cast formation, LBB, and tubular dilatation, with ATN devoid of focal coagulative necrosis visible as early as day 7. On day 21, only modest acute tubular damage and no interstitial fibrosis or tubular shrinkage were observed in the IRI-hpMSC group compared with day 28 in the IRI and IRI-PBS groups, where active tubular damage remained with localized atrophy (Figure 9 and Supplementary Materials Figure S4).

On day 28, significant RIS (renal injury score) differences were observed between IRI-hpMSCs and both IRI (*p* < 0.05) and IRI-PBS (*p* < 0.05). RIS, cast formation, and IFTA were significantly lower in the cell group than in the IRI and IRI-PBS groups. The details of the renal histology results are provided in the Supplementary Materials. The findings indicated that hpMSCs mitigated the injury to renal morphology in both the early and late phases.

## 3. Discussion

The potential of human placental mesenchymal stromal cells (hpMSCs) to prevent the initial cascade of kidney fibrosis and death was demonstrated by reducing initial kidney damage and improving kidney function during both the early and late phases of AKI.

CKD is considered a continuation of the same pathological mechanisms that lead to inadequate repair after AKI, including apoptosis and necrosis, pro-inflammation and anti-inflammation, and repair or fibrosis [17,48,49,50,51,52,53,54,55,56]. Thus, it is essential to find a treatment that decreases inflammation and prevents fibrosis [8,9,11] to stop the progression of AKI to CKD.

The mechanism by which mesenchymal stem cells (MSCs) promote renal function and reduce damage is still not completely understood. Researchers have argued that their protective and regenerative properties are primarily due to the paracrine mechanism [20,21,22,57]. However, studies have not provided evidence of the long-term survival of MSCs in vivo [22]. Several studies have shown the limited engraftment of MSCs into kidney tissue and their differentiation into epithelial and endothelial cells [23,24,25]. The most likely mechanism is related to the protective properties of stem cells that initiate repair through immunomodulation, proliferation, protection against oxidative stress, apoptosis, stimulation of neovascularization, and promotion of epithelialization processes, which ultimately result in reduced kidney tubular damage, prevention of renal glomerulosclerosis and fibrosis, and regeneration of tubules [18,26,27,28,29,30,32,33,34,35,36,37,38,40,41,42,43,44,58,59,60,61,62,63,64]. A majority of studies have demonstrated that MSCs improve functional recovery [39,40,41,42,43,59,60,61,64,65,66,67]. Despite promising preclinical results, the lack of successful clinical translation remains a significant challenge [44,59,68,69]. Development of novel strategies to enhance kidney function is crucial for the advancement of advanced therapies. These strategies must take into account past challenges in translational sciences, cell production, quality control, and clinical trial design to guarantee that future treatments are both effective and safe. In a clinical study conducted by Swaminathan et al., the benefits of MSCs in treating AKI were not demonstrated [44]. The study’s design, with unbalanced study arms, and the uncertainty surrounding the cell product’s potency may have contributed to the study’s lack of success [69,70]. Our exploratory study approach emphasizes the importance of learning from past experiences and achieving success in translational research through multidisciplinary collaboration. To achieve success, it is essential to focus on valid hypotheses, reproducible data, appropriate preclinical models, accurate statistical analyses, the impact of organizational structures, academic incentives, government funding, clinical relevance, and the promotion of transparency and data sharing [71,72].

Insufficiently defined manufacturing processes can exacerbate the inherent diversity of donor tissue sources. Calcat-i-Cervera et al. conducted a study to assess the influence of harmonized culture conditions across various laboratories on the characteristics of MSCs and to understand the differences associated with their tissue sources. The research team emphasized the variability in the behavior and regenerative capabilities of MSCs. Our study aimed to establish an optimal and consistent manufacturing process by carefully selecting pre-qualified donors and optimizing manufacturing and quality control processes [44,61,62,73,74,75].

Placenta-derived stem cells may be a suitable source for cell therapy in clinical practice due to their accessibility, low immunogenicity, and lack of ethical concerns [27].

To ensure that hpMSCs are clinically effective, it is crucial that they are well characterized and meet regulatory standards. The inconsistencies in trial outcomes underscore the necessity for potency assays that align with the therapeutic mechanisms. We proposed an integrated approach that incorporates various bioassays and analytical methods to fully evaluate the multifaceted therapeutic potential of MSCs [76,77].

In the current investigation, we conducted non-clinical experiments under Good Manufacturing Practice (GMP) conditions to utilize a cell product with specific characteristics for translational applications in the conversion of acute kidney injury (AKI) to chronic kidney disease (CKD). Our manufacturing methods aimed to replicate conditions that would be used in the clinical environment and have already demonstrated the benefits of human placenta-derived mesenchymal stem cell (hpMSC) injection in prolonging survival, reducing kidney damage, and improving kidney function. Our findings contribute to a better understanding of the survival advantages offered by placenta-derived stem cells, which align with a study of similar length using a cisplatin-induced AKI model and amniotic fluid placental stem cells administered intravenously. In the cell group, all mice survived until the end of the study, whereas fatalities began in the control group on day 5. By day 7, the survival rate in the group treated with cells was 56%, dropping to 33% by day 31, while no mice in the control group survived at these time points [78]. The improved survival rate observed in our study may be attributed to various factors, including differences in the kidney injury model, the types of cells used, and the dosages administered. Rota et al. injected a higher dose of 5 × 10^5^ cells/mouse, which is approximately 10 times the dose per weight that we used, but they administered the cells systemically. When cells are administered systemically, they can reach other parenchymal organs, thus requiring a higher effective dose. Different authors have reported therapeutic doses of experimental stem cell products in kidney injury models to range from 5 × 10^5^ cells to 5 × 10^6^ cells per rat [79,80,81]. In our study, we used a total of 6 × 10^5^ cells per animal, which was administered locally over two kidneys. Assuming a paracrine effect as the sole mechanism of action, 6 × 10^5^ cells correspond to a minimal dose used by others. We hypothesized that a non-inferior effect would be observed due to the higher concentration of cells in the damaged tissue.

Urea and creatinine are metabolic byproducts that are commonly used to evaluate renal function in humans. In our study, we found that the kidneys maintained normal diuresis volume and exhibited better renal function in the cell group. This was indicated by lower serum creatinine levels on day 3, which normalized more rapidly on day 7 in comparison to both control groups. Additionally, a lesser increment of urea nitrogen levels on days 3 and 7 in the cell group returned to the norm more rapidly. Creatinine levels in the urine remained normal in the hpMSC group after IRI but decreased in both control groups. Urinary urea levels also normalized in the IRI-hpMSC group but remained decreased in the IRI group on day 28. When interpreting the observed trends, it is essential to consider that death-censored estimates should be viewed as favoring control groups. Other researchers have also reported similar results, with decreased creatinine and/or urea nitrogen levels in the blood compared to the control group in short- and long-term AKI models [28,32,63,74,78]. In a kidney injury model with gentamicin conducted by co-authors using skeletal muscle-derived stem/progenitor cells (MDSPCs) in rats, no statistically significant difference in diuresis volume was observed between the cell and control groups [82]. To the best of our current knowledge, no other studies have investigated the impact of stem cells on diuresis volume. Our research demonstrated that hpMSCs can restore creatinine clearance more rapidly than the control groups. Furthermore, we observed an intriguing finding: creatinine clearance was higher in the IRI group than in the IRI-hpMSC group. We attributed this increased creatinine clearance and higher urine creatinine, as compared to the healthy control group on day 28 in the IRI group, to hyperfiltration resulting from AKI. In a study by Ashour et al., MSCs significantly improved creatinine clearance compared to the cisplatin-induced AKI group [74]. Our findings indicate that MSCs can improve kidney function, as measured using conventional markers. Alterations in electrolyte levels can lead to tubular injuries. The fractional excretion of potassium and sodium remained stable in the cell group. In addition, higher fractional potassium was observed in the IRI-PBS group compared to the IRI group, and this outcome could be attributed to additional intervention in the IRI-PBS group. To the best of our knowledge, no other studies with stem cells on electrolytes in blood and urine have been conducted.

Cells reduce structural damage to the kidney and prevent chronic injury, as shown by reduced tubular dilatation, ATN, cast formation, LBB, and IFTA. These results were confirmed using H&E, PAS, PSR, and MT staining. Our study supports the findings of others, who also noticed a similar positive effect on kidney morphology [32]. In the current study, serum creatinine normalized on day 28, but structural renal damage persisted on day 28 only in the control groups. Interstitial fibrosis, tubular atrophy, and total histological damage scores were significantly lower in the cell group than in the control group. Based on observations of other researchers, chronicity is expected to continue increasing with time [83,84].

Our research had several limitations. First, we employed both male and female animals in our experiments. The sex of the animals may have influenced the occurrence and outcome of acute kidney injury (AKI) and conversion of AKI to chronic kidney disease (CKD). Animal studies have demonstrated that female animals are less likely to develop AKI due to the nephroprotective effects of estradiol [85], whereas male animals are more likely to progress from AKI to CKD due to increased oxidative stress [86]. However, human studies have yielded conflicting results depending on the cause of AKI. Unlike preclinical studies, some clinical studies have shown that AKI progresses to CKD more frequently in females [87]. AKI is also more likely to occur in females after cardiac surgery, which is consistent with our non-clinical model [82]. These articles do not allow us to determine whether sex had an impact on our study, and the limitations of this study should be assessed separately in a dedicated laboratory. The results of these studies provide evidence of the potential beneficial effects of cell therapy to varying degrees in different sexes.

Second, the control and index arms employed different timelines, which were dictated by practical circumstances. The study was designed to be a 3-week-long study to observe differences in AKI reversal and AKI-related survival, as well as differences in the transition to CKD, if present. With respect to AKI reversal and survival, a three-week period was deemed more than adequate. In contrast, for the purpose of observing CKD development, it has been reported that a duration of at least two weeks is sufficient to detect chronicity [83,84]. The control-group animals were observed for a longer-than-one-week period, which may have conferred some advantage to the control group, although not to the same extent as was observed in terms of the structural damage, specifically the interstitial necrosis and tubular atrophy scars (see Figure 9d). After conducting an additional analysis, it was discovered that, on day 21, the median interstitial necrosis and tubular atrophy in the IRI-hpMSC group was approximately 0% percent, as compared to approximately 3% in the IRI-PBS group and around 10% in the IRI group. The two-week difference in the time periods led to a smaller mean difference that was reported by Kim et al., that is, approximately two times (from around 12% to around 24% in the area of positive MT staining per high-power field). We can conclude that hpMSCs reduced damage to kidney morphology in the early and later stages, thus protecting the kidneys from chronicity [28,29,30,32,33,34,35,36,37,63,70,75].

Thirdly, the small sample size in our study’s control groups resulted in a high level of variability, which is common in any AKI lesion. Despite this limitation, we identified a statistically significant advantage by comparing medians instead of means. The variability in the biomarker data observed in the control groups, particularly at the early AKI time points, did not diminish the overall benefit of active treatment demonstrated in the clinical and histological data, recovery from AKI, and survival.

## 4. Methods and Materials

### 4.1. In Vitro Methods

#### 4.1.1. Donor Eligibility

The placenta was collected from a healthy volunteer undergoing a Cesarean section, and the procedure was carried out with the approval from the Regional Bioethics Committee (Kaunas, Lithuania) (No. BE-2-105). The eligibility of the placental donors was confirmed in accordance with the current European Pharmacopoeia regulations. Only tissue samples from fully compliant donors were used for expansion. The potential of this starting material was thoroughly evaluated by determining its effect on the quality of the final hpMSC product.

The critical quality attributes of cell viability, population doubling, identity, and potency were assessed according to the specifications established for hpMSCs to minimize variability.

The performance of the starting material was confirmed at different stages. After digestion, the number of isolated cells (per mg of tissue) was measured, and after the isolation phase, the number of hpMSCs recovered from the cell factories was measured and compared to the initially seeded number of cells. The cells were then counted, and the population doubling (PD) was calculated. The results were compared with those obtained from previous donors.

#### 4.1.2. Characterization of hpMSCs

All the reagents and materials used in this study were of clinical grade. The hpMSCs were isolated and grown according to the Master Cell Bank’s Good Manufacturing Practice (GMP) protocols. The European Pharmacopoeia (Strasbourg, France) (EP) was followed for cell counting and the sterility and validation assays. The hpMSCs were incubated with primary antibodies against CD31-APC, CD45, CD73, CD90, and HLA-DR (BD FACSDiva) to assess the formation of osteoblasts, adipocytes, and chondrocytes. Senescence-associated β-galactosidase activity was also assessed. Karyotyping was performed using the G-banding method for chromosomal staining of the drug substance and product. The CFSE-labelled isolated mononuclear cells were activated with PMA/ionomycin. To investigate the immunomodulatory effects of hpMSCs, the secretion of inflammatory TNF-α and IL-6 was measured in cell-free supernatants using the PBMC assay. The in vitro methods are described in detail in the Supplementary Materials.

### 4.2. In Vivo Methods

#### 4.2.1. Animals

All animal procedures were authorized by the State Food and Veterinary Services (Vilnius, Lithuania). The study employed 8–12-week-old, 250–350 g male and female Wistar rats that were randomly allocated to all groups. A total of 3–10 rats were included per endpoint, resulting in a total of 43 rats for physiological analyses and 47 rats for survival analyses (Table S2). Prior to the surgical procedure, the animals were housed in an acclimatized room and fed a standard diet and water for 24 h.

#### 4.2.2. AKI to CKD Model

**Figure 10:**
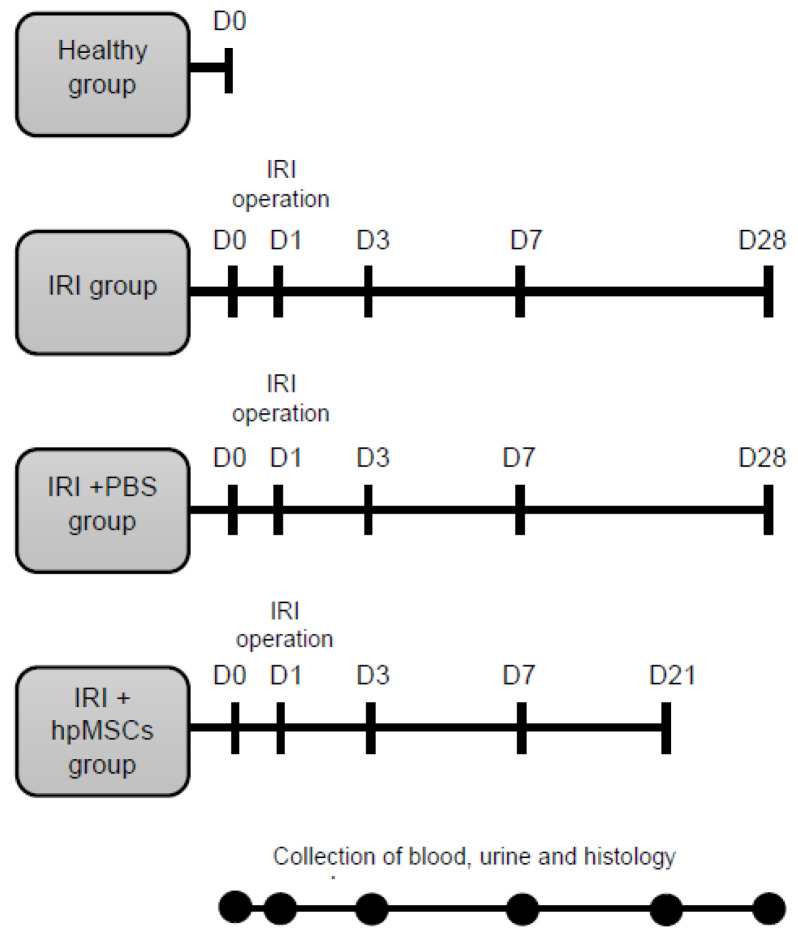
Experimental study design flowchart. Rats were randomly divided into four groups: healthy (healthy animals), IRI (IRI only), IRI-PBS (IRI plus PBS injection), and IRI-hpMSCs (IRI plus hpMSCs injection). The hpMSC injection was administered postoperatively after the release of the clamps at D1. Blood, urine, and tissue samples were collected on D0 (24 h before IRI) on days 3, 7, and 21–28 of the experiment.

The ischemia-reperfusion injury (IRI) model was employed to induce AKI. A graphical representation of the results is shown in Figure 10.

The experimental design included the (a) control group (healthy control), (b) untreated group (IRI), (c) IRI group receiving phosphate buffer solution (IRI-PBS), and (d) IRI group receiving human placental mesenchymal stem cells (IRI-hpMSCs).

The animals were premedicated and subjected to bilateral renal artery and vein occlusion with atraumatic microvascular clamps for 60 min under aseptic conditions. A single injection of placental stem cells (3 × 10^5^) was administered to the corticomedullary region of each kidney immediately after removal of the clamps. Retrograde cell leakage was observed in the preparatory phase, and the slowed speed of injection constantly minimized the leakage to negligible levels. The methods used to induce AKI are detailed in the Supplementary Materials.

Blood, urine, and tissue samples were collected to assess the renal function and tissue damage. Blood, urine, and tissue samples were collected on day 0 (24 h prior to IRI) and on days 3, 7, and 21–28 after IRI (using an automatic biochemistry analyzer Selectra Junior (Vital Scientific, Spankeren, The Netherlands). Urine samples were collected over a 24 h period using metabolic cages. The levels of creatinine, urea, potassium, and sodium were analyzed in both serum and urine samples. Creatinine clearance (CCr), fractionated potassium (${FEK}^{+})$, and fractionated sodium (${FENa}^{+})$ levels were calculated [88]. The method of calculating survival based on histological censoring is described in detail in the Supplementary Materials.

#### 4.2.3. Renal Histology

Kidney tissue samples were fixed in 10% buffered formalin and embedded in paraffin for subsequent analysis (National Pathology Center, Vilnius, Lithuania). The tissues were stained with hematoxylin and eosin (H&E) and periodic acid–Schiff (PAS) for light microscopic examination. The evaluation of acute tubular necrosis (ATN), dilatation, cast formation, loss of brush border (LBB), tubulitis, leukostasis, interstitial fibrosis, and tubular atrophy (IFTA) was conducted in 10 randomly selected, non-overlapping fields of each section, using the following grading system: 1, 0–35%; 2, 36–70%; and 3, 71–100%. The grading adhered to the standardized HESI (Health and Environmental Sciences Institute (HESI)/PSTC (ILSI, The International Life Sciences Institute) histopathology lexicon, as agreed upon by the relevant authorities (Melnikov et al., 2002) [89]. The degree of tubular injury was assessed by calculating the percentage of affected tubules. The percentage of tubular injuries was determined for each field as follows:

$$Renal\;injury\;score\;(RIS)\;\%\;=\;\left(\frac{Number\;of\;injured\;tubules}{Number\;of\;whole\;tubules}\right)$$

Masson’s Trichrome (MT) and Picro-Sirius Red (PSR) kit was used for visualization and collagen staining of tissues for semiquantitative assessment of IFTA. The histological slides were digitized at 20× magnification (0.5 µm per pixel) using an Aperio^®^ AT2 DX scanner (Leica Aperio Technologies, Vista, CA, USA).

#### 4.2.4. Statistical Analysis

Statistical analyses were performed using SPSS Statistics version 17.0 and GraphPad Prism 7.04 software. All groups had a sample size of at least three unless otherwise specified. Each batch underwent a single expansion, and duplicate measurements were performed on the same sample. The results are presented as the mean ± standard deviation for individual hPSCs donors and as an average across all three donors to account for inter-patient variability. Data were assessed for normal distribution using the Shapiro–Wilk test. Parametric data (hPSCs data) were analyzed using one-way ANOVA with Tukey’s post hoc multiple comparison test. Non-parametric data (clinical data) were analyzed using the median test. The results are presented as medians (horizontal lines). Survival was assessed using the curve comparison test. Statistical significance between the experimental groups was determined using a *p*-value < 0.05, as indicated by the asterisk (*).

## 5. Conclusions

The findings of the present investigation demonstrate the capacity of hpMSCs to reduce the progression of acute kidney injury to chronic kidney disease by mitigating initial kidney structural damage and enhancing renal function. In our study, we utilized a potency assay to estimate the mechanism of action of hpMSCs in kidney repair during the early stages.

## Data Availability

The data used to support the findings of this study are available from the corresponding author upon reasonable request.

## Supplementary Materials

The following supporting information can be downloaded at https://www.mdpi.com/article/10.3390/ijms25179647/s1.
