# Supplementary material for: Prevention of Transition from Acute Kidney Injury to Chronic Kidney Disease Using Clinical-Grade Perinatal Stem Cells in Non-Clinical Study
Source: Int J Mol Sci. 2024 Sep 6;25(17):9647. doi: 10.3390/ijms25179647 (PMC11394957; doi:10.3390/ijms25179647)
Supplement: Supplementary file 1 [file ijms-25-09647-s001.zip › ijms-3097899-supplementary.pdf]

Supplementary Materials

Methods and Materials

Donor eligibility

Placenta was collected from a healthy volunteer undergoing a Caesarean section. The procedure was carried out with appropriate ethical clearance and was approved by the Bioethics committee (No.: BE-2-105).

The eligibility of placental donors was confirmed according to current European Pharmacopoeia (EP) regulations. After obtaining written informed consent to donate tissues, a questionnaire assessing the risk of blood-borne infections, comorbidities, and used medications was completed. Blood serum NAT test results were negative for Hepatitis B and C, Human Immunodeficiency Virus I/II, and T. Pallidum.

**Test**	**Specifications**	**Donor 1**	**Donor 2**	**Donor 3**
Cell viability	More than 80%	97%	97%	95%
Population doublings	Less than 15	12	13	12
Identity markers	Positive expression > 95%<br>Negative expression < 1%	98%<br>0.3%	97.6%<br>0.5%	97.1<br>0.5
Inhibition potency	1:2,5 hpMSCs:PBMCs <50%	24%	33%	31%

Supplementary Table S1

hpMSCs isolation and proliferation methods

The placenta was processed in clean room facilities, employing a strict quality control approach that monitors personal and environmental in-process controls for microbiological, viral, cell count, and viability according to local law requirements. The tissues were then processed in a cleanroom. The cells were harvested and cryopreserved at 1 × 10^{6} cells/mL in cryopreservation medium (CryoStor CS10, BioLife Solutions). To collect human pluripotent stem cells (hpMSCs), the amniotic and chorionic layers were removed, and placental tissue was cut into 2-4 mm pieces. To isolate hpMSCs, placental membranes were removed from the placental body by blunt dissection and washed in Dulbecco's phosphate-buffered saline (DPBS; Gibco) to eliminate blood. After extensive washing in PBS containing 0.1% gentamicin, the tissue was digested for 2 h at 37°C in 0.1% collagenase (NB6, Nordmark) and DNase solution (Pierce nuclease, Thermo Fisher). After ACK lysis, the suspension was passed through a 150um cell strainer and rinsed. The cells were resuspended in DMEM containing 10% fetal bovine serum, plated in a tissue culture flask (Cell Factories, Corning), and incubated at 37°C in a humidified environment containing 5% CO_2. The medium was replaced after two days and every three days thereafter. After 8 to 10 days, sub-confluent cells were harvested with trypsin analog (TrypLE, Thermo Fisher) and reseeded at 1000 cells/cm^2. hpMSCs at passage four (drug prototype) were used in the present study.

Quality Controls of Final Product

Cell counts were evaluated using a Neubauer chamber or an automated cell counter (NucleoCounter NC-202). An automated cell counting approach was verified against the reference manual cell counting method of the EP 2.7.29. The cell count and viability were determined using trypan blue to discriminate

dead cells using a manual counting approach. Verification of automated cell counting based on accuracy, specificity, and precision. In each growth cycle, the following equation was used to determine the number of population doubling (PD): population doubling time = ln (2) × culture time [h/day]/ ln (final cell harvest number/initial cell number seeded). Cumulative PD (cPD) was computed by aggregating the PDs of each passage. The doubling time (dT) was calculated by dividing the number of days necessary for complete cell growth by cumulative PD.

Culture media (BD Bactec Plus) was used to analyze cell supernatants for aerobic and anaerobic microorganisms and fungi, following the EP 2.6.27, to evaluate the sterility of the process. These guidelines outline the recommended procedures for sterility testing in cell-based products. Adherence to established standards ensures the reliability and consistency of the results. Throughout the manufacturing process, the conditioned media was included as a sterility reference sample. These reference samples served as controls and were used as a baseline for comparison to ensure that any potential contamination was reliably detected and identified. By including conditioned media as reference samples at different stages of the process, deviations or issues in sterility testing can be promptly addressed and resolved.

A strict mycoplasma testing schedule was established and implemented for each passage before freezing and after thawing. A validated real-time PCR assay (VenorGeM qEP, Minerva) was used to detect mycoplasma contamination following EP 2.6.7. The qPCR assay is rapid, robust, and sensitive, making it an ideal tool for identifying mycoplasma contamination. The qPCR assay utilized a TaqMan (Thermo Fisher) based approach, which is a well-established and widely recognized method for detecting specific nucleic acid sequences with high accuracy and precision. The assay validation process ensured that it consistently produced accurate and reliable results, thereby providing confidence in its performance. The cell supernatant and 1×10^6 cells were examined because mycoplasma contamination can occur in various forms and locations. Testing both the cell supernatant and a substantial number of cells significantly increases the likelihood of detecting mycoplasma contamination, ensuring that no hidden or overlooked contamination compromises the quality of hpMSCs.

A kinetic chromogenic quantitative Limulus Amoebocyte Lysate (PTS2005F, Charles Rivers) test was performed for endotoxin analysis, following the guidelines specified in the EP 2.6.14. The LAL test enables real-time reaction monitoring between the LAL reagent and the endotoxins. An automated spectrophotometer, the Endosafe nexgen-PTS, was used to streamline the process and ensure accuracy. This advanced instrument automates measurements, reduces human error, and provides rapid and reliable results. The preparation process included conditioned media containing cells as the endotoxin reference samples. The reference samples served as controls, providing a baseline for comparison during endotoxin analysis. Analysing conditioned media at different stages of the manufacturing process can promptly identify and address any variations or potential issues in endotoxin levels.

Immunophenotypic analysis

For immunophenotypic characterization, 1×10^6 hpMSCs were washed with FACS buffer containing PBS supplemented with 1% bovine serum albumin (BSA; Sigma). The cells were then incubated in the dark at room temperature for 30 min with the following fluorochrome-conjugated primary antibodies (all from BD): anti-CD31-APC (#558068), anti-CD45-FITC (#561865), anti-CD73-PE (#562817), anti-CD90-PE (#562385), and anti-HLA-DR-FITC (#555811). The cells were washed with FACS buffer prior to acquisition using a fluorescence-activated cell sorting device (BD FACS Aria III).

All the reagents were used in accordance with the manufacturer's instructions. The relative fluorescence intensity of the cells was then analyzed using BD FACS Diva 8.0.1 software (BD Immunocytometry Systems, Germany).

Multilineage differentiation

To induce osteogenic differentiation, a density of 5×10^3 cells/cm2 was used to seed the cells in 12-well plates. They were cultured in Stem Pro Osteogenesis Differentiation medium (Thermo Fisher,) for 3

weeks. The medium was changed every three days, and the onset of osteoblast formation was assessed by analysing the expression of Alizarin Red. Next, the cells were treated with the StemPro Adipogenic Differentiation Kit (Thermo Fisher,) for two weeks to induce adipogenic differentiation. Medium changes were carried out every three days, and the onset of adipocytes was evaluated using Oil Red O (Sigma) staining. A chondrogenic micromass was generated from cultures by seeding 5-µL droplets of cell solution in the center of a 6-well plate. After 14 days in culture, the medium was removed from the culture vessel, fixed with formaldehyde, rinsed, and stained with Alcian Blue to evaluate chondrogenic differentiation.

Peripheral blood mononuclear cells (PBMCs) proliferation assay

A peripheral PBMCs proliferation inhibition assay was performed. Briefly, 500.000 isolated mononuclear cells were stained with 5(6)-carboxyfluorescein diacetate N-succinimidyl ester (CFSE) and incubated overnight at 37°C. The basal fluorescence level of cell proliferation was measured using a cell sorter (BD FACS Melody). Three different ratios of hpMSCs:PBMCs coculture were evaluated: 1:10, 1:5, and 1:2.5. CFSE-labelled PBMCs proliferation was activated with PMA/ionomycin according to the manufacturer's protocol. After five days of co-culture, the supernatants were collected and PBMCs proliferation was evaluated by measuring the fluorescence using a cell sorter (BD FACS Melody). The relative fluorescence intensity of PBMCs proliferation was subsequently analyzed (BD FlowJo, v.9). Gating was used to remove debris and to select single cells for CFSE staining. Chondrocytes were used as negative controls in the assay.

TNF-α and IL-6 ELISA assay

To investigate the immunomodulatory effects of hpMSCs, the secretion of inflammatory TNF-α and IL-6 was measured in cell-free supernatants from PBMC assays. Enzyme-linked immunosorbent assay was performed on five samples per condition according to the manufacturer's protocol (Biotechne, USA). The optical density was read at 450 nm using a microplate reader (Infinite M Nano, Tecan). A standard curve was plotted as the relative optical density of each standard solution against the concentration of the standard solution. The respective gene concentrations in the samples were interpolated from a standard curve.

β-galactosidase activity assay

A senescence-associated beta-galactosidase activity assay was done up till 8th passage using a colorimetric test (Cell signalling Technology, #9860S). The cells were cultured in 6-well plates at a density of 1×10^5 cells/well until 80% confluency was reached. After fixation with 2% PFA and 0.2% glutaraldehyde, cells were stained according to the manufacturer's instructions. Cells were inspected and the generation of senescent cells was evaluated. The overall percentage of stained cells in the cell population was averaged across the five fields.

Karyotyping study

In vitro karyotyping was performed using the G-method of chromosome staining for the final hpMSCs preparation. Drug substances and products were evaluated separately for P2 and P4, respectively. MSCs were cultured in T25 flasks until reaching 70-80% cell confluence Then, 60 µl KaryoMAX™ Colcemid™ Solution in PBS (10 µg/ml; ThermoFisher Scientific) was added to the flask and incubated at 37°C for 2 h. MSCs were detached using 1 mL of prewarmed TrypLE™ Select Enzyme 1X (ThermoFisher Scientific), lysed with hypotonic solution (0.075 M KCL) prewarmed for 30 min at 37°C, and fixed in Methanol: Acetic acid (3:1) solution. Metaphase slides were made by dropping cells on glass slides, incubating at 60 °C for at least 16 h, and then Giemsa staining. According to the Committee for Advanced Therapies of the European Medicines Agency recommendations at least 20 metaphases and an exclusion limit of two metaphases (10%) with clonal chromosomal aberration were analyzed. Chromosomes were described according to the International System for Human Cytogenomic Nomenclature.

AKI to CKD (ischemia-reperfusion injury) ModelRats were randomly divided into four groups: healthy (healthy animals), IRI (untreated) (IRI only), IRI-PBS (control) (IRI plus PBS injection), and IRI-hpMSCs (CellS) (IRI plus hpMSCs injection). Blood, urine, and tissue samples were collected on D0 (24 h before IRI), on days 3, 7, and day 21-28 of the experiment.

Animals were premedicated with inhalational and intraperitoneal anesthesia was used during surgery (sevoflurane 3%, buprenorphine at a dose of 0.05 mg/kg). Animals were operated under aseptic conditions, both the left and right renal pedicles were identified, and the renal artery and vein were occluded with atraumatic microvascular clamps on each side (bilateral) to achieve complete ischemia of the corresponding kidneys and minimize damage to the adventitia layer. Ischemia was visually verified by the change in kidney color to dark purple. The clamps were released 60 min after occlusion. A single placental stem cell injection (3 × 10 (5)) was administered in the corticomedullary region of each kidney after removing the clamps. Reperfusion was confirmed by the recovery of the initial color of the kidney.

Glomerular filtration rate (GFR) was calculated by to the creatinine clearance (CCr):

$$C_{Cr} = \frac{(U_{Cr} \times V_{U/24h})}{(S_{Cr} \times 24 \times 60)}$$

Fractionated potassium (FEK^{+}) was calculated using the following formula:

$$FEK^{+} = \frac{U_{K^{+}} \times S_{Cr}}{S_{K^{+}} \times U_{Cr}} \times 100$$

Fractionated sodium ($FENa^{+}$) was calculated using the following formula:

$$FENa^{+} = \frac{U_{Na^{+}} \times S_{Cr}}{S_{Na^{+}} \times U_{Cr}} \times 100$$

All urine values were standardized using creatinine in the urine. 24 hours urine creatinine, urea, and potassium levels were calculated using the following formula:

$$U_{change} = U_{X} - U_{y}$$

Where x stands for end time-point and y - for starting time-point.

In vivo methods

Animals

All animal procedures were authorized by the State Food and Veterinary Services. The study employed 8–12-week-old, 250-350 grams male and female Wistar rats, which were randomly allocated to all groups. 3-10 rats were included per endpoint (a total of 43 rats for physiological analyses, 47 rats for survival analyses). (Table S2) Prior to the surgical procedure, animals were housed in an acclimatized room and fed a standard diet and water for 24 h.

Groups	Healthy animals	IRI (n=20)					IRI-PBS (n=23)					IRI-hpMSCs(n=10)					TOTAL
Days of decapitation female – F) (male – M)	D0	D0-D3	D4-D7	D8-D14	D15-D28	Total (D0-D28)	D0-D3	D4-D7	D8-D14	D15-D28	Total (D0-D28)	D0-D3	D3-D7	D8-D14	D15-D28	Total (D0-D28)	Total (D0-D28)
The number of rats for survival analysis female – F) (male – M)	5 (F-3; M-2)	4 (F-2; M-2)	5 (F-2; M-3)	2 (F-2; M-0)	4 (F-4; M-0)	15 (F-10; M-5)	5 (F-5; M-0)	3 (F-3; M-0)	5 (F-2; M-3)	4 (F-1; M-3)	17 (F-11; M-6)	3 (F-2; M-1)	3 (F-1; M-2)	0	4 (F-3; M-1)	10 (F-6; M-4)	47 (F-30; M-17)
The number of physiology analyses female – F) (male – M)	5 (F-3; M-2)	5 (F-3; M-2)	5 (F-2; M-3)	0	5 (F-5; M-0)	15 (F-10; M-5)	5 (F-5; M-0)	4 (F-4; M-0)	0	4 (F-1; M-3)	13 (F-10; M-3)	3 (F 2; M-1)	3 (F-1; M-2)	0	4 (F-3; M-1)	10 (F-6; M-4)	43 (F-29; M-14)
The number of histological analyses of survived rats and decapitated female – F) (male – M)	5 (F-3; M-2)	4 (F-2: M-2)	5 (F-2; M-3)	0	4 (F-4; M-0)	13 (F-8; M-5)	5 (F-5; M-0)	3 (F-3; M-0)	0	4 (F-1; M-3)	12 (F-9; M-3)	3 (F-2; M-1)	3 (F-1; M-2)	0	4 (F-3; M-1)	10 (F-6; M-4)	40 (F-26; M-14)
The number of deaths female – F) (male – M)	0	4 (F-2; M-2)	0	1 (F-0; M-1)	0	5 (F-2; M-3)	6 (F-4; M-2)	0	0	0	6 (F-4; M-2)	0	0	0	0	0	11 (F-6; M-5)

Supplementary Table S2. Physiological and Survival data.

Results

Diuresis

The IRI-hpMSCs group kidneys retained a normal diuresis volume during the entire 28-day study period. Kidney retained normal diuresis throughout the 28-day study period in cells group, and a significant deterioration in the IRI group compared to the cell treated group (*P* = 0.005) during the same period. (Figure 9) The first three days of the study were significantly superior in the cell-treated group, compared to the IRI (*P* = 0.002) and IRI-PBS (*P* = 0.002) groups, as manifested by no polyuria stage evident increase in diuresis. In addition, a lower diuresis volume change between D3 and D7 was evident in the treated IRI-hpMSCs group than in the IRI group (*P* = 0.02), reflecting the preventive mode of action of the perinatal stem cell prototype. (Supplementary Figure S1) This study showed sustained diuresis in the treatment group.

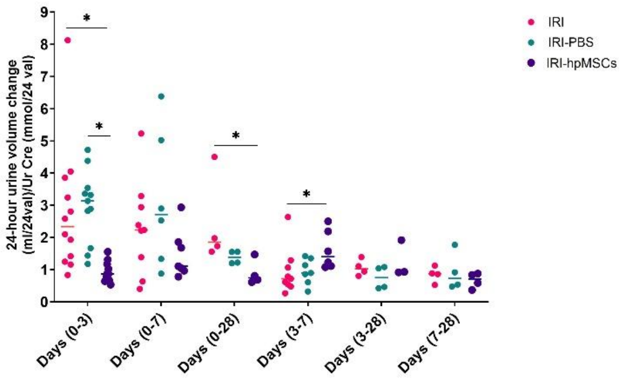

Supplementary Figure S1. Kidneys retain normal diuresis in the cell group. Total diuresis volume change over 24 h in the experimental groups. IRI-D (0-3) vs IRI-hpMSCs-D (0-3); IRI-PBS-D (0-3) vs IRI-hpMSCs-D (0-3); IRI-D (0-28) vs IRI-hpMSCs-D (0-28); IRI-D (3-7) vs IRI-hpMSCs-D (3-7), * $P < 0.05$.

Creatinine and urea levels in serum and urine

Serum creatinine levels in the IRI group increased significantly (*P*= 0.003) on day 3 relative to those in the healthy control group, but then began to drop (*P* = 0.002) on day 7 and returned to normal (p = 0.018) by day 28. The IRI-PBS group showed an increase in creatinine levels (*P* = 0.002) on day 3 relative to the healthy control group, which decreased (*P* = 0.003) on day 7 owing to enhanced diuresis and returned to normal (*P* = 0.018) on day 28. The IRI-hpMSCs group showed an increase in serum creatinine levels on day 3 relative to the healthy control group, but the increase was not statistically significant (*P* = 0.46). The levels returned to normal (*P* = 0.46) on day 7 and remained normal on day 28. (Figure 9)

The purpose of this study was to examine the effects of IRI-hpMSCs on urine creatinine levels. (Supplementary Figure S2) The IRI-hpMSCs group demonstrated normal creatinine levels in urine throughout the duration of the experiment, with no statistically significant differences between D0 and D3 (*P* = 0.5), D7 (*P* = 0.38) and D28 (*P* = 0.24), different than in IRI (D3, D7, D28) and IRI – PBS (D3) groups. In addition, the urinary creatinine levels were higher (better kidney concentration function) in the IRI-hpMSCs group on day 3 than in the IRI group (*P* = 0.007). However, creatinine levels in urine were higher in the IRI group on day 28 (p=0.005). On day 28, the higher urinary creatinine level in the IRI group than in the healthy control group was attributed to hyperfiltration due to acute renal injury. In the IRI group, urine creatinine levels were considerably lower on D3 than on D0 (*P* = 0.008), began to rise on D7 and rising further on D28 (*P* = 0.018). The IRI-PBS group demonstrated a statistically insignificant decrease in urine creatinine levels on D3 compared to D0 (*P* = 0.85), with diuresis beginning to increase on D7 (*P* = 0.64) and normalizing by D28 (*P* = 0.018). In conclusion, the IRI-s group consistently exhibited normal urinary creatinine levels.

In a study comparing creatinine secretion in three experimental groups, the IRI group showed a substantial decrease from day 0 to day 3 compared with the IRI-hpMSCs group (*P* = 0.03). From days 3 to 7 in the same groups, the drop was even more pronounced, with a statistically significant increase in urine creatinine in the IRI (*P* = 0.01) and IRI-PBS (*P* = 0.02) groups compared to that in the IRI-hpMSCs group. Lastly, a greater increase in urinary creatinine from day 3 to day 28 was observed in the IRI (*P* = 0.047) and IRI-PBS (*P* = 0.047) groups than in the IRI-hpMSCs group. This represents a higher magnitude of recovery from D3 to D28 in the control groups (IRI and IRI-PBS groups).

The creatinine clearance in the IRI-hpMSCs group decreased considerably on day 3 compared to that in the healthy control group (*P* = 0.03), increased on day 7 (*P* = 0.03), and increased subsequently on day 28, but did not reach the level of the healthy control group (*P* = 0.016). In the IRI-PBS group, creatinine clearance decreased considerably on day 3 relative to that in the healthy control group (*P* = 0.002) and remained lower on days 7 (*P* = 0.03) and 28 (*P* = 0.016). On day 3, the creatinine clearance in the IRI group was considerably lower than that in the healthy control group (*P* = 0.016). Creatinine clearance in the IRI group remained severely lowered on day 7 (*P* = 0.002) and increased on day 28 (*P* = 0.002). On day 7, a non-significant trend was observed, with the IRI-PBS group (surviving rats) having a lower creatinine clearance than the IRI-hpMSCs group (*P* = 0.66). Creatinine clearance on day 28 was significantly greater in the death-censored IRI group than in the IRI-hpMSCs group (*P* = 0.005). On day 28, the higher creatinine clearance in the IRI group than in the healthy control group was interpreted as hyperfiltration, a hallmark of acute kidney injury. In conclusion, the data indicate that hpMSCs can restore creatinine clearance more rapidly than the control groups.

The IRI-hpMSCs serum urea levels increased less dramatically on day 7 and reverted to normal levels more quickly. On day 7, serum urea levels in the IRI-PBS group were significantly higher (*P* = 0.014) than those in the IRI-hpMSCs group. On day 3, there was no statistically significant difference in urea levels between the IRI-PBS and IRI-hpMSCs groups (*P* = 0.47); however, a clear trend towards lower serum urea was evident in the cell group. In the IRI-hpMSCs group, urea levels increased on day 3 (*P* = 0.47), but to a lesser extent than in the IRI and IRI-PBS groups, remained stable on day 7 (*P* = 0.47), and increased insignificantly by day 28 (*P* = 0.3). In conclusion, the increase in urea in the cell group on days 3 and 7 was less pronounced than that in the IRI and IRI-PBS groups, and it reverted to normal levels more rapidly.

A numerical reduction in 24-hour urine urea levels in all groups by day 3 was evident in this study. On day 28, despite this decrease, the IRI group had a lower urine urea concentration than the IRI-hpMSCs group. The return of urinary urea was significantly higher in the IRI-hpMSCs group than in the IRI group (*P* = 0.005). The 24-hour urine urea in the IRI group was significantly reduced compared to baseline on day 3 (*P* = 0.001) and remained lower on days 7 (*P* = .001) and 28 (*P* = 0.03). The IRI-PBS group exhibited a substantial decrease in urine urea on day 3 compared to baseline (*P* = 0.03), as well as a decrease on day 7 (*P* = 0.16); however, levels reverted to normalize on day 28 (*P* = 0.2).In the IRI-hpMSCs group, urinary urea decreased considerably compared to baseline on day 3 (*P* = 0.001), remained reduced on day 7 (*P* = 0.001), and reverted to baseline on day 28 (*P* = 1.0). In conclusion, the results imply that urine urea remained low in the IRI group on day 28, whereas it returned to normal in the IRI-hpMSCs group.

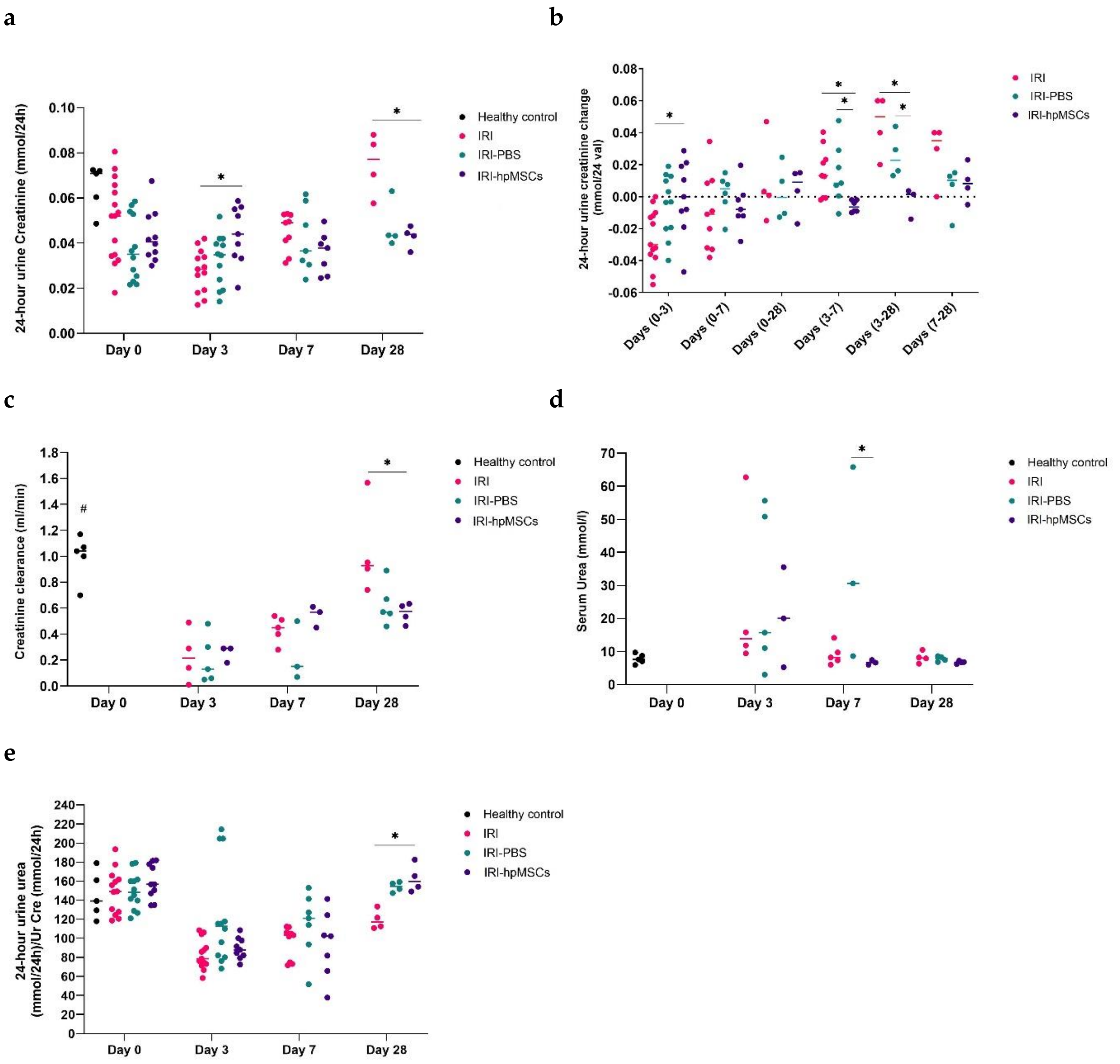

Supplementary Figure S2. Cells attenuate the increase in serum creatinine levels, retain creatinine levels in the urine, and restore creatinine clearance faster. (a) Urine creatinine level over 24 h in the experimental groups. IRI-D3 vs IRI-hpMSCs-D3; IRI-D28 vs IRI-hpMSCs-D28, IRI-D0 vs IRI-D3; IRI-D7 vs IRI-D28, * $P < 0.05$. (b) Urine creatinine change over 24 h in the experimental groups. IRI-D (0-3) vs IRI-hpMSCs-D (0-3); IRI-D (3-7) vs IRI-hpMSCs-D (3-7); IRI-PBS-D (3-7) vs IRI-hpMSCs-D (3-7); IRI-D (3-28) vs IRI-hpMSCs-D (3-28); IRI-PBS-D (3-28) vs IRI-hpMSCs-D (3-28), * $P < 0.05$. (c) Creatinine clearance levels in the experimental groups. Healthy control vs. IRI-D3, D7 and D28; Healthy control vs. IRI-PBS-D3, D7, D28; Healthy control vs. D3, D7, and D28, Δ $P < 0.05$; IRI-D28 vs. IRI-hpMSCs-D28 * $P < 0.05$. Kidneys more rapidly improved recovery of serum urea level and restored urine urea level in cells group. (d) Serum urea levels in experimental groups. IRI-PBS-D7 vs IRI-hpMSCs-D7, * $P < 0.05$. IRI-hpMSCs-D0 vs D3, D7, and D28, ns. (e) Urine urea for 24 h in the experimental groups. IRI-D28 vs. IRI-hpMSCs-D28; IRI-D0 vs. D3, D7, and D28; IRI-PBS-D0 vs. D3; IRI-hpMSCs-D0 vs. D3, D7 * $P < 0.05$.-hpMSCs-D0 vs D28, ns.

Electrolytes levels in serum and urine

The kidneys maintained normal serum potassium levels throughout the study period. (Supplementary Figure S3) In the IRI-PBS group, serum potassium levels increased significantly ($P = 0.028$) compared to the IRI-hpMSCs group on day 3. In the IRI group, potassium levels increased on day 3 ($P = 0.3$) and then declined on day 7 ($P = 0.53$), before returning to normal on day 28 ($P = 0.3$). On day 3, potassium levels were maintained in the IRI-hpMSCs group ($P = 0.47$) until day 21 ($P = 0.3$). The study revealed that there were no cases of hyperkalemia in the cell group.

The kidneys in the cell group maintained normal urine potassium levels throughout the investigation period. The urine potassium levels in the IRI-hpMSCs group remained identical to baseline from day 3 (*P* = 0.81) to the last day of the study (*P* = 0.18), while those in the control group were significantly increased. The results of the study revealed that the IRI-PBS group had significantly higher urine potassium levels on day 3 than the IRI-hpMSCs group (*P* = 0.004). Analysis of the IRI group revealed that the urine potassium level increased considerably on day 3 compared to baseline (*P* = 0.04), continued to increase on day 7 (*P* = 0.02), and returned to baseline on day 28 (*P* = 0.17). The IRI-PBS group demonstrated a considerable increase in urine potassium levels on day 3 relative to day 0 (*P* = 0.001), a minor reduction on day 7 (*P* = 0.001), and an increase on day 28 (*P* = 0.015). Urine potassium levels in the IRI-hpMSCs group remained identical to baseline from day 3 (*P* = 0.81) up to the last day of the study (*P* = 0.18).

Urine potassium changes from day 0 to day 3 were significantly higher in the IRI-PBS group than in the IRI-hpMSCs group (p=0.0006).

Fractional excretion of potassium is a direct indicator of kidney tubular damage. in the IRI group was considerably higher than that in the healthy control group (*P* = 0.003) on day 3. This increase persisted on day 7 (*P* = 0.002), but by day 28, it had returned to the level of the healthy control group (*P* = 0.73). The fractionated potassium level in the IRI-PBS group was considerably higher than that in the healthy control group on day 3 (*P* = 0.002), reduced significantly on day 7 (*P* = 0.028), and declined further but remained elevated on day 28. (*P* = 1.0). The fractionated potassium level in the IRI-PSC group was lower than that in the other groups relative to the healthy control group on day 3 (*P* = 0.028), decreased on day 7 (*P* = 0.028), and decreased but remained elevated on day 28 (*P* = 1.0). The fractional potassium level on day 7 was substantially greater in the IRI-PBS and IRI groups than in the IRI-hpMSCs group (*P* = 0.014 and *P* = 0.028, respectively). In addition, the fractional potassium level on day 28 was substantially greater (*P* = 0.005) in the IRI-PBS group than in the IRI-hpMSCs group.

A comparison of fractionated sodium on day 3 between the IRI-hpMSCs and IRI groups (surviving rats) revealed a clear trend toward higher fractionated sodium levels in the IRI group, but the difference was not statistically significant (*P* = 0.27). A similar trend toward higher fractionated sodium was found in the IRI-PBS group (surviving rats) compared to the IRI-hpMSCs group on day 3, although the difference was not statistically significant (*P* = 0.47). In addition, fractionated sodium in the IRI-hpMSCs group increased less than that in the healthy control group (P = 0.028) on day 3 and remained unchanged on days 7 (P = 0.028) and 28 (*P* = 0.099). On day 3, fractionated sodium levels in the IRI group were considerably higher than those in the control group (*P* = 0.003). This increase dropped on day 7 (*P* = 0.002) and continued to decline until day 28 (*P* = 0.003). The fractionated sodium concentration in the IRI-PBS group was considerably higher than that in the healthy control group on day 3 (*P* = 0.058) but significantly lower on days 7 (*P* = 0.47) and 28 (*P* = 0.099). In conclusion, the findings showed that the fractionated sodium levels in the examined cell group remained reasonably steady.

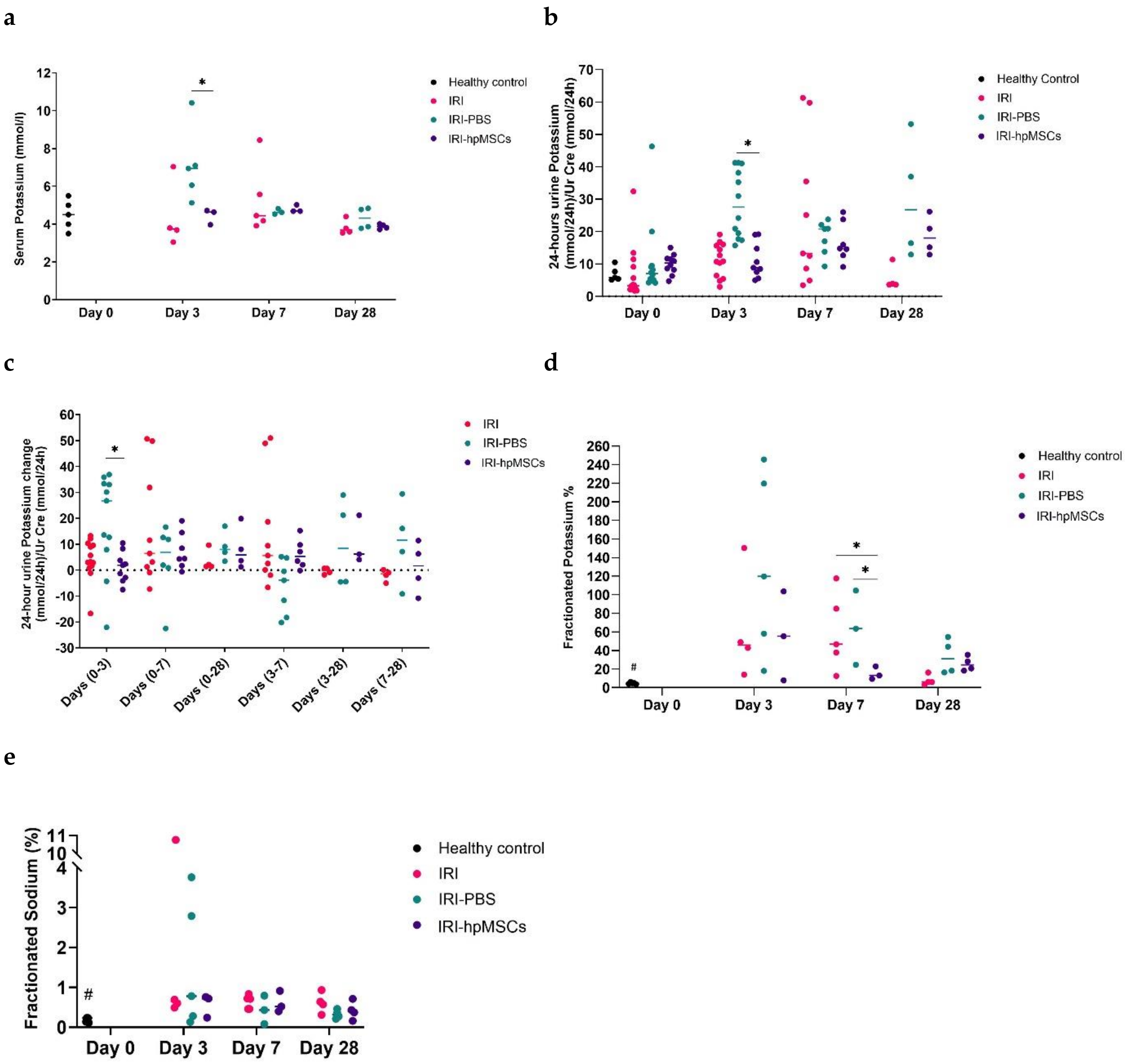

Supplementary Figure S3. Kidneys maintain normal serum potassium and sodium level in cells group. (a) Serum potassium levels in the experimental groups. IRI-PBS-D3 vs IRI-hpMSCs-D3, * $P < 0.05$. IRI-hpMSCs-D0 vs D3, D7, and D28, ns. (b) Urine potassium over 24 h in the experimental groups. IRI-PBS-D3 vs. IRI-hpMSCs-D3; IRI-PBS-D0 vs. D3, D7, and D28; IRI-hpMSCs-D0 vs. D7, * $P < 0.05$. IRI-hpMSCs-D0 vs D3, D28, and ns. (c) Urine potassium change over 24 h in the experimental groups. IRI-PBS-D (0-3) vs IRI-hpMSCs-D 0-3), * $P < 0.05$. (d) Fractionated potassium levels in the experimental groups. IRI-D7 vs. IRI-hpMSCs-D7; IRI-PBS-D7 vs. IRI-hpMSCs-D7; IRI-PBS-D28 vs. IRI-hpMSCs-D28 * $P < 0.05$; Healthy control vs. IRI-D3 and D7; Healthy control vs. IRI-PBS-D3 and D7; Healthy control vs. D3 and D7, # $P < 0.05$. (e) Fractionated sodium levels in the experimental groups. Healthy controls vs. IRI-D3, D7, and D28, # $P < 0.05$.

Renal histology

In addition, the loss of brush border effect (sign of chronicity) was significantly reduced in IRI-hpMSCs compared to both the IRI ($P = 0.005$) and IRI-PBS ($P = 0.005$) groups on day 28. (Supplementary Figure S4) Tubular dilatation was lower in IRI-hpMSCs group compared to IRI ($P = 0.005$) and IRI-PBS ($P = 0.005$) groups on day 28. Interstitial necrosis and tubular atrophy were significantly lower on day 28 in the IRI-hpMSCs group than in the IRI group ($P = 0.005$).

a

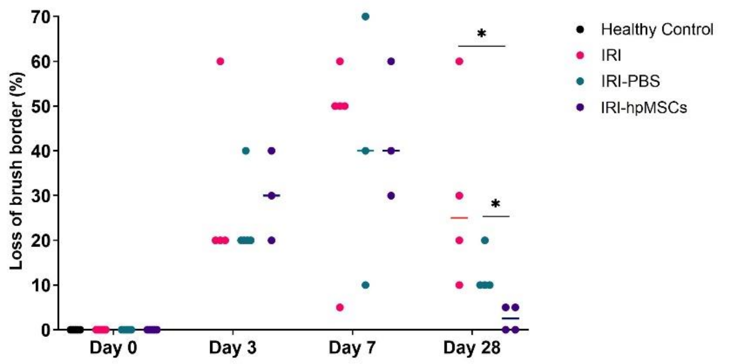

b

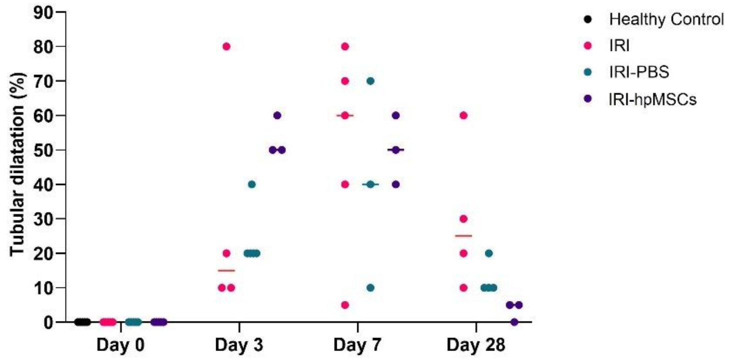

Supplementary Figure S4. Cells reduce structural damage to the kidneys and prevent chronic injury. (a) Loss of brush border in the experimental groups. IRI-D28, IRI-PBS-D28 vs IRI-hpMSCs-D28, * $P < 0.05$. (b) Tubular dilatation in the experimental groups. IRI-D28, IRI-PBS-D28 vs IRI-hpMSCs-D28, * $P < 0.05$.
